# Selective and Sensitive OECT Sensors with Doped MIP-Modified GCE/MWCNT Gate Electrodes for Real-Time Detection of Serotonin

**DOI:** 10.1021/acsomega.4c10918

**Published:** 2025-01-24

**Authors:** Amin Mehrehjedy, Jack Eaton, Kan Tang, Saroj Upreti, Aries Sanders, Vincent LaRoux, Xiaodan Gu, Xuyang He, Song Guo

**Affiliations:** † Department of Chemistry and Biochemistry, School of Mathematics and Natural Sciences, The University of Southern Mississippi, Hattiesburg, Mississippi 39406, United States; ‡ School of Criminal Justice, Forensic Science, and Security, The University of Southern Mississippi, Hattiesburg, Mississippi 39406, United States; § School of Polymer Science and Engineering, The University of Southern Mississippi, Hattiesburg, Mississippi 39406, United States; ∥ Department of Chemistry, The University of Arkansas − Fort Smith, Fort Smith, Arkansas 72913, United States

## Abstract

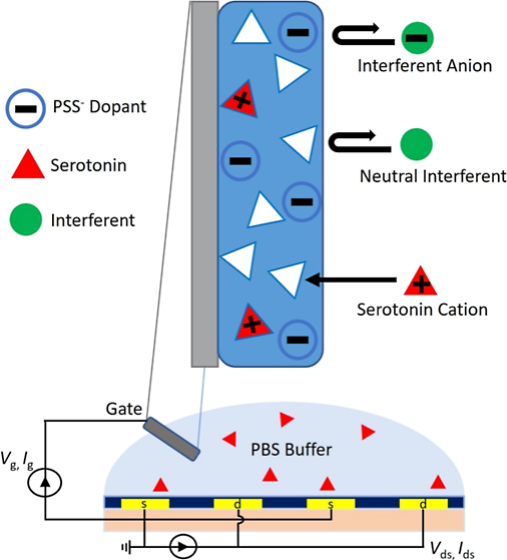

Organic electrochemical transistors (OECTs) represent a promising platform for biosensing applications in aqueous environments, including the sensitive detection of neurotransmitter molecules, such as serotonin (SE). Conventional methods for SE detection, such as HPLC and ELISA, are time-consuming and expensive. Electrochemical sensors, while sensitive and cost-effective, often struggle with real-time detection and selectivity issues due to interference from similar biomolecules, such as dopamine (DA), ascorbic acid (AA), and uric acid (UA). These interferents are particularly challenging for the OECT detection because they are easier to oxidize than SE on the gate electrode. Molecularly imprinted polymer (MIP) has gained increasing interest in electrochemical analysis, providing a cost-effective method for the selective detection of various analytes by creating matching cavities in the polymer film. Herein, a glassy carbon/multiwall carbon nanotube (GCE/MWCNT) gate electrode was modified by a PSS^–^doped overoxidized molecularly imprinted polymer (DOMIP) layer in an OECT sensor. Characterizations by cyclic voltammograms (CV), electrochemical impedance spectroscopy (EIS), scanning electron microscopy (SEM), Raman spectroscopy, and wide-angle X-ray scattering (WAXS) demonstrate an improved conductivity of the gate electrode due to DOMIP modification. The resulting GCE/MWCNT/DOMIP sensor demonstrated a low detection limit of 0.31 μM for SE in real-time measurements, comparable to that of the GCE/MWCNT sensor. However, the GCE/MWCNT sensor showed little selectivity toward SE. In addition to the SE-templated cavities, the DOMIP gate electrode modification leveraged the electrostatic interactions between the negatively charged PSS^–^ dopant and the positively charged SE molecules to achieve a higher sensitivity toward SE compared to other negatively charged or neutral interferents in the concentration range of 0.31 μM - 3.1 μM. These findings suggest that combined with the GCE/MWCNT gate electrode, the doping strategy used in DOMIP-modified OECT sensors could provide a low-cost way for the selective and real-time monitoring of SE in complex biological samples without the usage of noble-metal electrode or expensive antibodies, which is potentially suitable for a large-scale medical diagnosis.

## Introduction

Organic electrochemical transistors (OECTs) are a group of thin-film transistors with a configuration like that of the field-effect transistor OFET. OECTs are made of source, drain, and gate electrodes but rather than a dielectric, an electrolyte is between the gate and the source/drain (S/D) electrodes.^1^ Conducting polymers, such as poly(3,4-ethylenedioxythiophene) polystyrenesulfonate (PEDOT:PSS), have been used as active channels on the S/D electrode, facilitating the transport of both ions and charge carriers.^2^ Ions can be exchanged between the active channels and the electrolyte, thereby changing the doping state and conductivity of the active channels, and modulating the drain current (*I*_d_).^3^ This makes OECTs a promising candidate for the detection of analytes, such as neurotransmitters,^4,5^ proteins,^6−8^ and other biomolecules^9−11^ in aqueous media.

The OECT can operate in either depletion or accumulation modes. PEDOT:PSS is a p-type doped conductor, and the OECT that uses it as the active channel operates in the depletion mode. PEDOT:PSS acts as a conductor of holes upon applying bias to S/D and before applying gate voltage (*V*_g_) due to the charge balance between the PEDOT polymer chain and sulfonate PSS^–^ anion. After applying a positive *V*_g_, cations such as Na^+^ are repelled from the electrolyte to the active channel. This will dedope the PEDOT:PSS, and consequently decrease in drain current as *V*_g_ increases, effectively switching off the device.^12^

Serotonin (SE) is a pivotal neurotransmitter that regulates a variety of physiological processes, highlighting the medical importance of its precise measurement. An abnormal low level of SE correlates with conditions, such as depression and anxiety, while a high level of SE is associated with autism.^13^ Measurement of SE in body fluids has significant medical relevance to screen medical conditions, such as carcinoid tumors and liver cell regeneration.^14,15^ Conventional methods for detecting serotonin (SE), such as HPLC, ELISA, radioimmunoassay (RIA), and GC–MS, are time-consuming, costly, and require specialized expertise and expensive instrumentation.^16^

Various electrochemical sensors have been developed for detecting SE, utilizing methods, such as cyclic voltammetry (CV), chronoamperometry (CA), differential pulse voltammetry (DPV), and square wave voltammetry (SWV).^17,18^ These methods employ different working electrodes, including noble-metal-based electrodes and modified carbon-based electrodes. For instance, the glassy carbon electrode (GCE) can be modified with multi walled carbon nanotubes MWCNTs (GCE/MWCNT). These non-noble-metal electrochemical sensors are cost-effective, easy to use, and sensitive but cannot measure the change of SE concentration in real-time.^16^ Although the detection mechanism of SE using OECTs differs from that of traditional electrochemical sensors, the oxidation of SE on the gate electrode highlights the potential of the GCE/MWCNT as a promising candidate for gate electrodes in OECT-based sensing platforms.

So far, there have been only a few examples of sensors for real-time SE measurements, primarily based on fast scanning cyclic voltammetry (FSCV)^19,20^ and aptamer-based sensors.^21,22^ Electrochemical sensors utilizing aptamers to modify the gold working electrode have been reported for real-time measurement of SE.^22^ These sensors demonstrate high selectivity and sensitivity and can be designed as portable devices for point-of-care applications. However, the production of aptamers is a labor-intensive and costly process, requiring multiple rounds of screening for high-affinity aptamers, which makes the scale-up production of aptamer-based sensors challenging.^21,22^ Similarly, FSCV-based sensors, while effective, needs complicated instrumentation and is not suitable for large-scale production.^20,23^ The lack of extensive reports on real-time detection of SE,^16,24^ combined with the need for a simple and cost-effective method for scaling up SE sensor production, highlights the importance of new research in this field.

Recently, our group reported an OECT sensor operating in the depletion mode using PEDOT:PSS as active channels for the real-time detection of dopamine (DA). The mechanism of DA detection by the OECT sensor is as follows: the oxidation of DA on the gate electrode generates a redox faradic current, causing a shift in the applied *V*_g_ to a higher effective gate voltage (*V*_g,eff_), and induces a shift in the drain current, which serves as the signal for the detection of DA.^4^ A conductive overoxidized molecularly imprinted polymers (OMIP) made of polypyrrole (PPy) was deposited on a platinum (Pt) gate electrode using a simple electropolymerization method, which successfully increased the selectivity of the Pt gate toward DA and effectively blocked ascorbic acid (AA).^4,9,10,25^ However, SE’s higher oxidation potential and susceptibility to interference from similar biomolecules posed significant challenges that could not be addressed with the previous configuration. Recently, Sasso et al. have added PSS^–^ to the MIP layer to increase the electrostatic interaction between the doped MIP layer and the charged target molecule in an electrochemical sensor.^26^ At physiological pH (7.4), SE is positively charged, while the interferants uric acid (UA) and ascorbic acid (AA) are negatively charged.^27^ Therefore, by leveraging the electrostatic interactions, doping the PPy-based MIP is a promising strategy to simultaneously improve the sensitivity and selectivity.

In this work, a gate-modified OECT sensor in the depletion mode is fabricated for the selective and sensitive detection of the SE. PEDOT:PSS is used as the active channel, and GCE/MWCNT is used as the gate electrode. Without the usage of noble metal (Au or Pt) gate electrode, the GCE/MWCNT-gated OECT exhibited high sensitivity for detecting SE. However, it also demonstrated even greater sensitivity toward interferents, including AA, UA, and DA, because SE is more difficult to oxidize. To extend the sensor’s capability for SE, we introduced poly(sodium 4-styrenesulfonate) (PSS^–^) as a dopant to PPy-based OMIP (DOMIP) on the GCE/MWCNT electrode using the electrochemical deposition method. A real-time SE detection limit of 0.31 μM by the GCE/MWCNT/DOMIP-gated OECT is obtained, and a high selectivity toward SE over UA, DA, and AA is demonstrated at low to medium concentrations.

## Experimental Section

### Materials

PEDOT:PSS pH1000 (1.3%) was purchased from Ossila, UK. Polystyrenesulfonate, dopamine hydrochloride, ascorbic acid, uric acid, and serotonin hydrochloride were purchased from Fisher Scientific. Dimethylformamide (DMF) was from Sigma-Aldrich. Pyrrole was purchased from Oakwood Chemical, Estill, SC. DI water was obtained from a Millipore water purification system. Electrolytes, such as disodium phosphate (Na_2_HPO_4_) and monosodium phosphate (NaH_2_PO_4_), were also purchased from Sigma-Aldrich. Multi-walled carbon nanotubes were purchased from MSE supplies. The concentrations of Na_2_HPO_4_ and NaH_2_PO_4_ were 10 and 1.8 mM, respectively, for the preparation of a PBS buffer solution (pH = 7.4). Prefabricated interdigitated electrodes (IDEs) were from NanoSPR, LLC, Chicago, IL. The channel length L of the IDEs is 20 μm, and the total channel width W is 20 mm (1 mm each ×20 pairs). The GCE electrode and electrode polishing kit were purchased from CH Instruments.

### Electrochemical Measurements

All electrochemical measurements were carried out on an electrochemical analyzer (CH Instruments, CHI660A) with a standard three-electrode system. The counter electrode was a platinum (Pt) mesh; the reference electrode was a Ag/AgCl (saturated KCl), and the working electrode was a glassy carbon electrode (GCE), and a modified GCE. Electrochemical impedance spectroscopy (EIS) was performed in a KCl solution with a 5 mM ferro/ferricyanide redox probe. The CV of the SE redox reaction in the presence of different GCE and modified GCE electrodes was conducted in a 0.1 mM SE and PBS electrolyte solution.

### Fabrication of GCE/MWCNT/DOMIP Gate Electrode

Prior to modification, the GCE electrode was polished with an alumina powder, rinsed with DI water, and dried with nitrogen. To fabricate the GCE/MWCNT, 10 mg of MWCNT was dispersed in 10 mL of DMF to obtain a 1 mg/mL slurry solution. Then, 10 μL of the prepared slurry was drop-cast onto the electrode and dried in an oven at 50 °C for 30 min to obtain GCE/MWCNTs.

The DOMIP film was prepared with the same three electrode setup in electrochemical measurements. To electropolymerize DOMIP, three cycles were swept between 0 and +1 V in a solution containing 10 mM pyrrole, 0.1 M PSS, and 1 mM serotonin hydrochloride, with a scan rate of 10 mV/s. After depositing the DMIP film, it was immersed in an ethanol solution for 1 h to remove the SE template. Then, the DMIP film was overoxidized (DOMIP) by using five cycles swept from 0 to 0.5 V in 0.5 M NaOH with a scan rate of 10 mV/s. The prepared electrodes were stored in DI water for the next steps. OMIP was prepared using the same method, replacing PSS^–^ with 0.1 M NaCl. The SEM measurements were performed in a Zeiss Sigma VP FEG-SEM instrument with a Thermo Scientific UltraDry EDS detector. A thin layer of gold was sputter-coated onto the samples prior to SEM imaging to enhance the conductivity.

### Source/Drain Preparation, OECT Device Fabrication, and Characterization

The previously reported method was used to make the S/D electrode.^4^ Briefly PEDOT:PSS was spin coated on interdigitated electrodes (IDEs) and annealed at 140 °C for 1 h. The transfer curves and real-time drain measurements of the OECT devices were conducted using a dual-channel Keithley 2612B source measure unit, controlled by the Keithley ACS software. The PBS buffer (pH = 7.4) was used as the electrolyte. Transfer curves were conducted with *V*_g_ swept between 0 and +1, while the source-drain voltage (*V*_ds_) was biased at −0.1 V by using different gate electrodes. For real-time measurements, *V*_g_ and *V*_ds_ were biased at −0.1 and 0.5 V, respectively, while different concentrations of SE were added. The standard deviation of the baseline before the addition of SE was used as the baseline noise. After each addition, any change in current higher than three times the baseline noise is considered a signal.

## Results and Discussion

**Figure 1:**
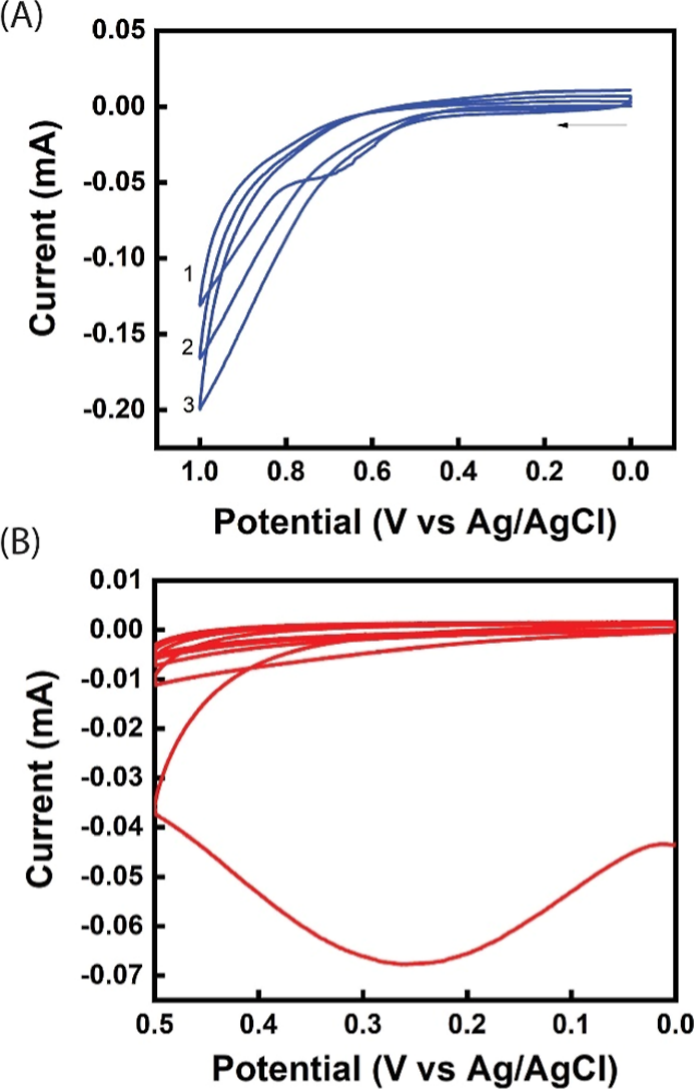
Cyclic voltammogram of (A) electrochemical polymerization of PPy-based DMIP film. The DMIP film was prepared by electropolymerization with three CV cycles between 0 and +1 V in a solution containing 10 mM pyrrole, 0.1 M PSS, and 1 mM serotonin hydrochloride, at a scan rate of 10 mV/s. (B) Overoxidation of PPy-based DMIP film. (DOMIP). The DOMIP film was obtained by overoxidizing the DMIP layer with five CV cycles from 0 to 0.5 V in 0.5 M NaOH at a scan rate of 10 mV/s.

Figure 1A shows the cyclic voltammogram (CV) of the electrochemical synthesis of DMIP. The oxidation peaks for SE and pyrrole appear around 0.6 and 0.95 V, respectively. The peak for SE is present only in the first peak. The gradual increase in the intensity of the pyrrole oxidation peak from the first cycle to the third cycle is evidence of the conductivity of the PPy film. After removal of the SE template using ethanol, overoxidation was performed to increase the surface area of the PPy film, further enhancing the interaction of SE with the DOMIP film, thus increasing the sensitivity of the DOMIP film. The CV of the overoxidation process is presented in Figure 1B, which shows a broad peak around 0.35 V for the first cycle and no significant peak for the remaining four cycles, indicating that most of the overoxidation occurred in the first cycle.

**Figure 2:**
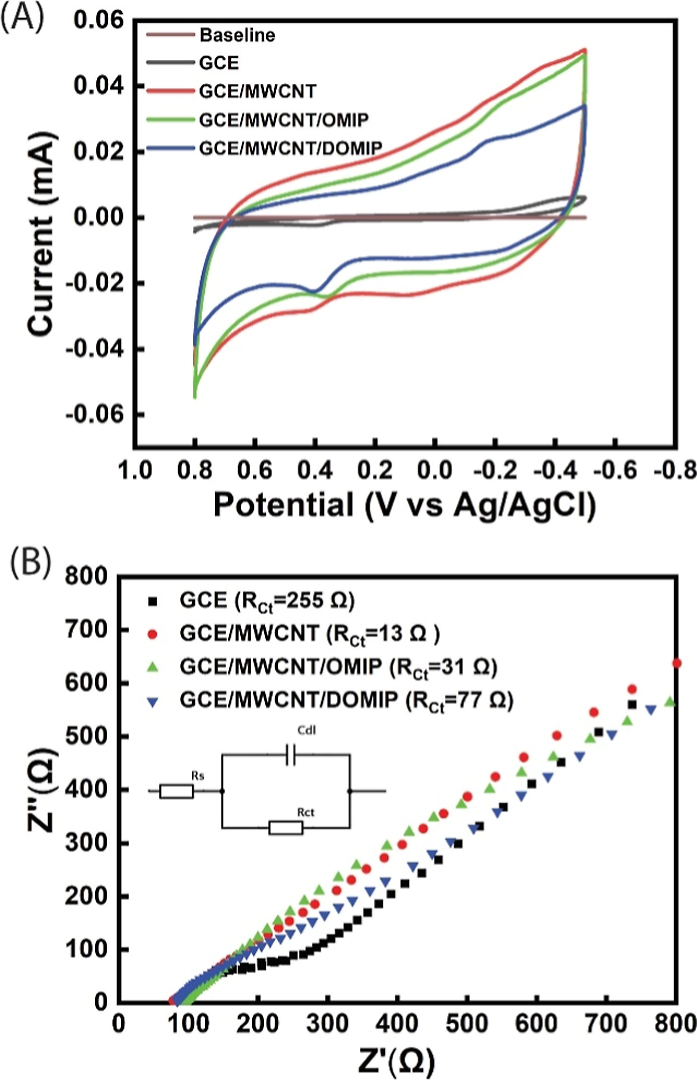
(A) CV of 0.1 mM SE redox reaction using GCE and GCE-modified electrodes in the PBS electrolyte. Baseline was measured in PBS without SE (B) EIS measurements of GCE and GCE-modified electrodes in the presence of 5 mM ferro/ferricyanide in 1 M KCl electrolyte.

Figure 2A shows the cyclic voltammograms (CVs) of the bare GCE and modified GCE electrodes in the presence of 0.1 mM SE. For the bare GCE, there is a very small oxidation peak for SE. Because the detection mechanism of the OECT sensor is based on redox reactions at the gate electrode, this result suggests that the bare GCE is not suitable as a gate electrode. However, for GCE/MWCNT, GCE/MWCNT/OMIP, and GCE/MWCNT/DOMIP, an oxidation peak with high current can be observed at around 0.40 V, and the absence of a reduction peak suggests the irreversible oxidation of serotonin. The higher current of the oxidation peak could be attributed to the increased surface area of the electrode and the increased number of oxidation reaction sites upon modification by MWCNTs, and the lower resistance of OMIP- and DOMIP-modified GCE/MWCNT electrodes, which enhance the faradaic current.^28^ The larger oxidation current observed at about 0.40 V on DOMIP than on OMIP suggests that the incorporated PSS^–^ dopants promote a higher number of SE molecules to undergo adsorption and oxidation on DOMIP, especially in the SE-selective cavities. These results demonstrate that modified GCE electrodes can be effectively used as gate electrodes and a *V*_g_ of 0.5 V is sufficient to establish the redox reaction at the gate electrode, resulting in a high sensitivity of the OECT sensor. A shift in the oxidation potentials of the modified gate electrodes show electrocatalytic oxidation of SE^29^

To study the differences in resistance of GCE and modified GCE electrodes, EIS measurements were recorded in the presence of the ferric/ferrocyanide redox couple for GCE and modified GCE electrodes, and the results are shown in Figure 2B. The Randle equivalent circuit was used to estimate the charge-transfer resistance on different GCE and modified GCE electrodes. As shown in the figure, the Randle circuit consists of electrolyte resistance (*R*_s_), double layer capacitance (*C*_dl_), Warburg impedance (*Z*_W_), and charge-transfer resistance (*R*_ct_). The results demonstrate that the *R*_ct_ values for the GCE are higher than those for modified GCE electrodes, with the former being around 255 Ω. In contrast, the modified GCE electrodes exhibit significantly lower *R*_ct_, specifically, 13 Ω for GCE/MWCNT, 31 Ω for GCE/MWCNT/OMIP, and 77 Ω for GCE/MWCNT/DOMIP, respectively. The lower *R*_ct_ of the modified GCE electrodes is attributed to the high surface area and high electrical conductivity of the MWCNT. Although PPy is also conductive, its conductivity is lower than that of MWCNT,^30^ resulting in a higher *R*_ct_ (Figure 2B).

**Figure 3:**
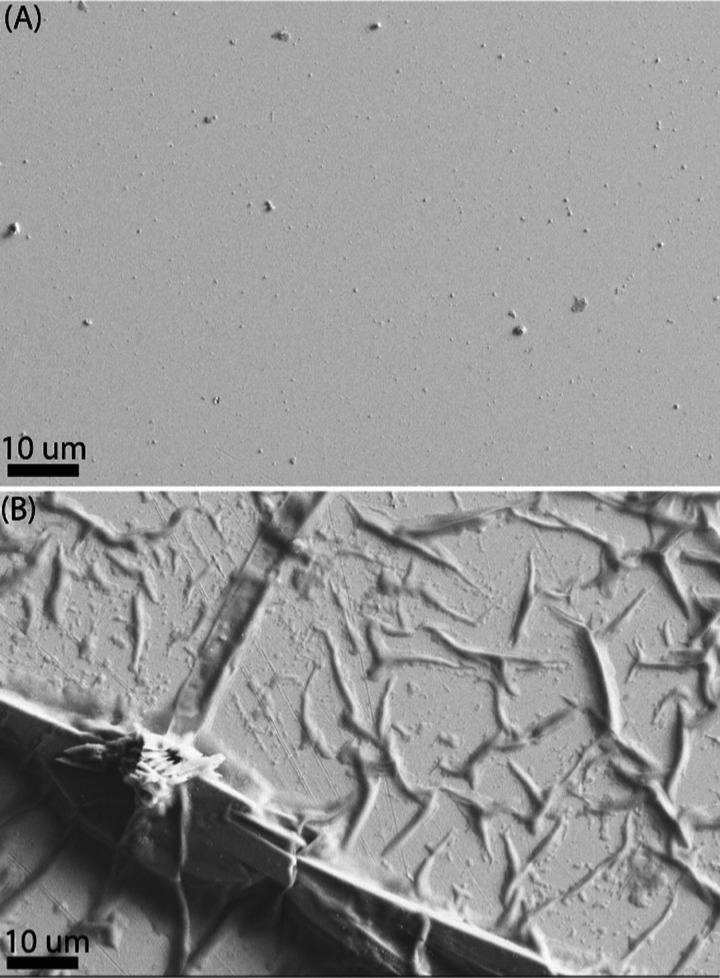
SEM images of (A) electropolymerized DMIP (3 CV cycles, 0 to 1 V) and (B) overoxidized DMIP and DOMIP (5 CV cycles, 0 to 0.5 V).

The morphology of the DMIP and DOMIP films was studied by using scanning electron microscopy (SEM), as displayed in Figure 3. The SEM images of the DMIP film show a uniform layer, suggesting that complete surface coverage was achieved. This is probably due to the low CV scan rate during the deposition of the PPy film, which allows sufficient time for the polymer film to fully cover the electrode surface. The SEM images of the DOMIP film reveal the formation of a dendritic structure resulting from the electrochemical etching of the MIP film in NaOH solution. This etching process increases the surface area and the number of available reaction sites for the oxidation of the SE, potentially enhancing the sensitivity of the OECT sensor.

**Figure 4:**
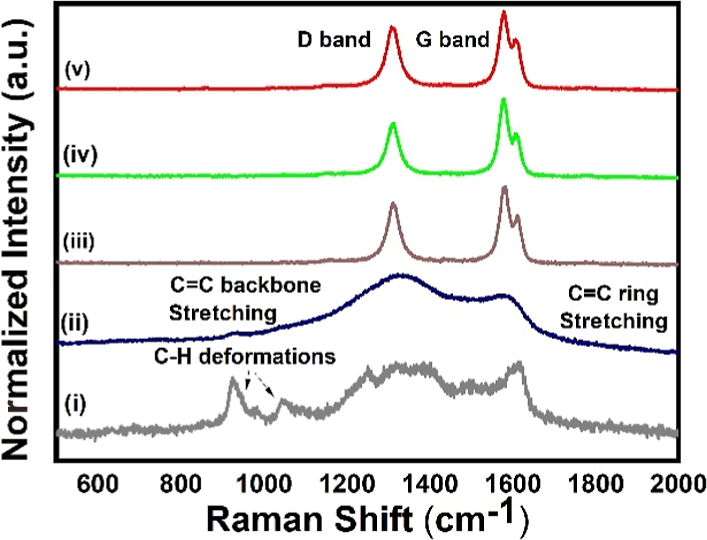
Raman spectra of (i) DOMIP, (ii) DMIP, (iii) MWCNT/DMIP, (iv) MWCNT/DOMIP, and (v) MWCNT.

Figure 4 displays the Raman spectra of PPy, overoxidized PPy, MWCNT, MWCNT/PPy, and MWCNT/overoxidized PPy. The Raman spectrum of PPy shows characteristic bands at 1572 and 1337 cm^–1^, which correspond to the C=C backbone stretching and the ring stretching vibrations of PPy, respectively. Upon overoxidation, the PPy spectrum exhibits a shift from 1580 to 1620 cm^–1^, and two new peaks emerge at 925 and 1047 cm^–1^, representing C–H in-plane deformation and out-of-plane deformation vibrations, respectively. These changes between the Raman spectra of PPy and those of overoxidized PPy are due to the treatment of the PPy film with NaOH, which increases defects in the PPy film. During the overoxidation process, the hydroxide ions attack the main chains of PPy, resulting in a reduced π-conjugation length and a transition to a quinoid structure. These changes could contribute to the enhanced affinity of the overoxidized film toward SE. Raman spectra of MWCNT show two prominent characteristic peaks: the D band at 1310 cm^–1^ and the G band at 1590 cm^–1^. The intensity of these peaks in MWCNTs is significantly stronger than those of PPy, which is a characteristic of MWCNT. Because there is an overlap between the peaks of PPy, overoxidized PPy, and MWCNT, and the intensity of these peaks in the MWCNT overwhelms those of PPy and overoxidized PPy, the Raman spectra of MWCNT/PPy and MWCNT/overoxidized PPy are similar to those of MWCNT.

**Figure 5:**
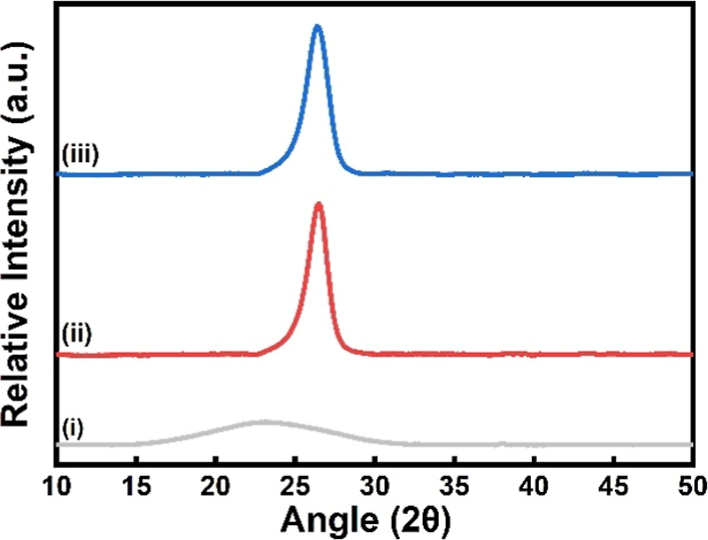
Wide-angle X-ray scattering (WAXS) of (i) DOMIP, (ii) MWCNT/DOMIP, and (iii) MWCNT thin films.

The wide-angle scattering patterns of the MWCNT, DOMIP, and MWCNT/DOMIP thin films are shown in Figure 5. The MWCNT peak observed at 2θ = 26° corresponds to the (002) graphite-like structure. A similar pattern is observed for the MWCNT/DOMIP thin film, attributed to the higher MWCNT content relative to that of DOMIP within the composite structure. For the bare PPy, a broad peak ranging from 2θ = 15° to 33° is observed, indicating the amorphous nature of the thin film.

**Figure 6:**
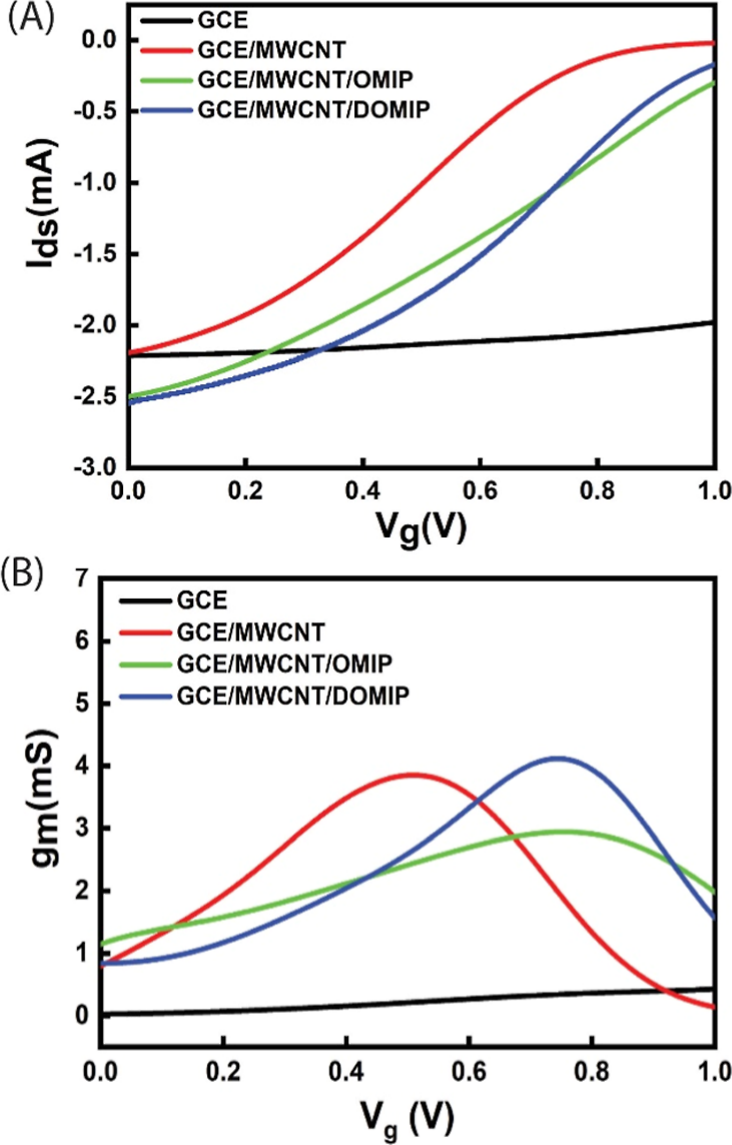
(A) Transfer curves and (B) transconductance *g*_m_ of OECTs as a function of gate bias (*V*_g_) of OECT device with bare GCE, GCE/MWCNT, GCE/MWCNT/OMIP, and GCE/MWCNT/DOMIP gated sensors.

As the mechanism of detection of SE is based on the oxidation of SE on the gate electrode, an appropriately high voltage should be applied to the gate electrode to ensure the oxidation of SE. Although a gate voltage (*V*_g_) higher than the oxidation potential of SE (0.4 V) is required to oxidize SE, a high transconductance (*g*_m_) is also necessary to ensure the high sensitivity of the sensor. In Figure 6A, transfer curves (*I*_d_ – *V*_g_) of the GCE and modified GCE electrodes resulting from transfer characteristic measurements are shown. A significant shift is observed between the transfer curves of GCE and modified GCE electrodes, indicating that a substantially higher *V*_g_ is required to turn off the OECT gated with GCE. This difference could be due to the differences in their *R*_ct_, where a higher *R*_ct_ in GCE leads to a lower effective gate voltage (*V*_g,eff_). Figure 6B shows the transconductance of different GCE and modified GCE-gated OECTs. *V*_gm_ is the gate voltage (*V*_g_) at which the highest transconductance is achieved. Transconductance for GCE-gated OECTs is low in the operating voltage range of 0–1 V. In contrast, modified GCE electrodes exhibit high transconductance in this range. The *V*_gm_ for GCE/MWCNT is 0.52 V, and for GCE/MWCNT/OMIP and GCE/MWCNT/DOMIP-gated OECTs, it is around 0.75 V. All modified GCE electrode-gated OECTs show decent transconductance at the oxidation potential of the SE (0.35 V). High *V*_g_ can damage the PEDOT:PSS film of the active channels, resulting in a noisy *I*_d_ current during real-time measurements. At *V*_g_ = 0.5 V, a voltage higher than the oxidation potential of the SE, the most stable current in real-time measurement was achieved and the GCE/MWCNT/DOMIP-gated OECT shows good transconductance. Therefore, for real-time measurements, a *V*_g_ of 0.5 V was applied to the gate electrode.

**Figure 7:**
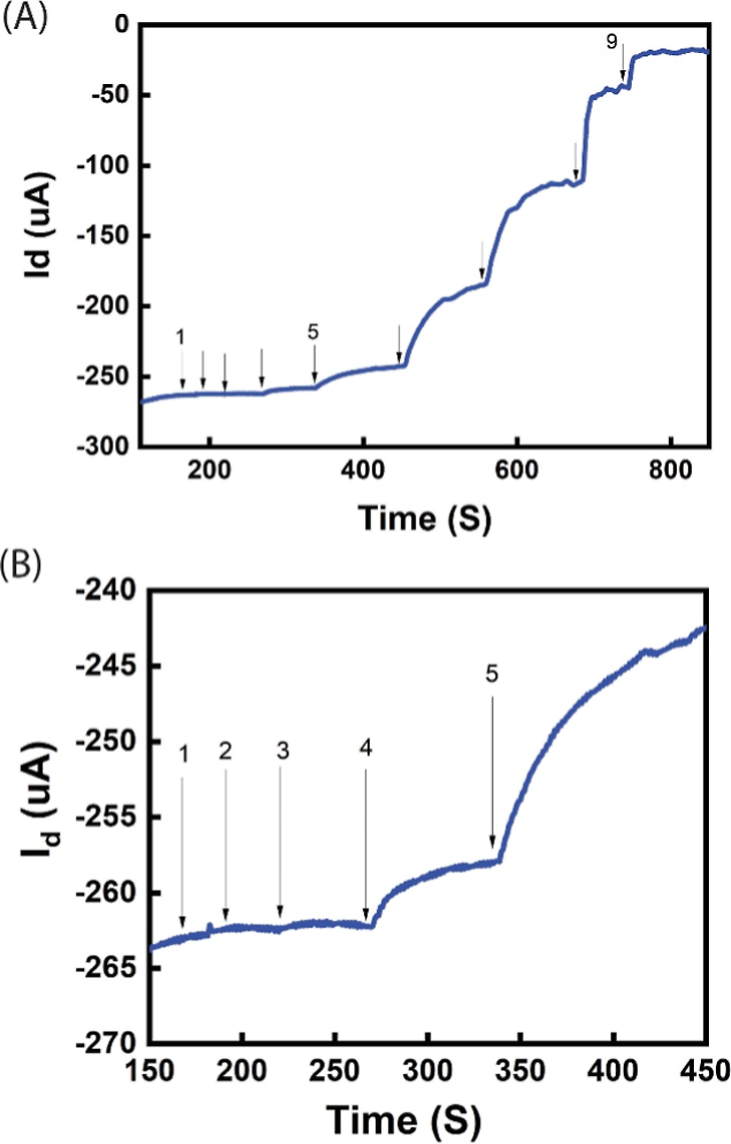
(A) Real-time trace of *I*_ds_ of OECT sensors with GCE/MWCNT/DOMIP gates upon sequential additions of SE in PBS buffer. The cumulative SE concentrations for these additions are 4.7, 27.7, 74.7 nM, 0.31, 0.77, 3.07, 7.78, 30.82, and 77.92 μM, respectively. (B) Real-time trace of *I*_ds_ of OECT sensors with GCE/MWCNT/DOMIP gates for the first five additions shown in (A).

Figure 7 illustrates the real-time measurements for the GCE/MWCNT- and GCE/MWCNT/DOMIP-gated devices, demonstrating a decrease in the magnitude of *I*_d_ after each addition of SE at varying concentrations. These nine consecutive additions are 4.7, 23, 47 nM, 0.23, 0.47, 2.35, 4.71, 23.5, and 47.1 μM, resulting in the cumulative concentrations of 4.7, 27.7, 74.7 nM, 0.31, 0.77, 3.07, 7.78, 30.82, and 77.92 μM, respectively. The *V*_g_ and the *V*_ds_ were biased at −0.1 V and +0.5 V, respectively. The observed step-like reduction in *I*_d_ serves as the detection signal for small biomolecules, including neurotransmitters. In the initial two additions of 4.7 and 23 nM, there was no significant change in *I*_d_ observed. The third addition of 47 nM sometimes resulted in a minor drop, but this was not consistent across all trials and is therefore not considered a detectable signal. A substantial change in *I*_d_ was observed after the fourth addition (0.23 μM), establishing a cumulative concentration of 0.31 μM as the limit of detection (LOD) for the sensor. This LOD is comparable to the LOD (0.1 μM) of real-time SE measurement by fast-scan cyclic voltammetry (FSCV).^19^ Although aptamer-based methods provided better LOD, the overall device fabrication process in this work is much simpler and more cost-effective.^21^ Subsequent additions at higher concentrations (0.47, 2.35, 4.71, 23.5, and 47.1 μM) resulted in step-like decreases in current. The magnitude of these decreases grew with the first three additions but then diminished as the sensor approached saturation. Similar to SE, a step-like drop in *I*_d_ current can be observed for interferents, such as AA, UA, and DA (Figure S1).

The normalized ending current response (NCR) can then be calculated using the following formula and plotted as a function of the accumulated analyte concentrations.
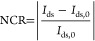


**Figure 8:**
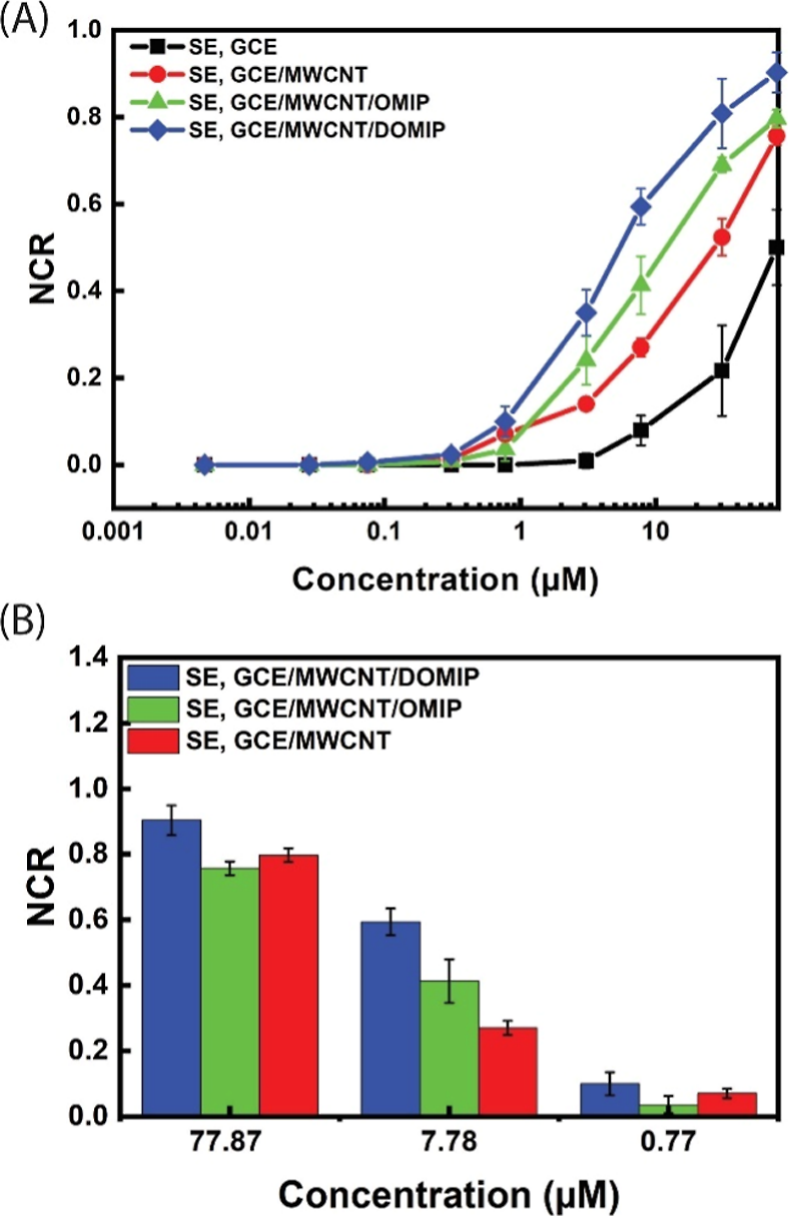
(A) NCRs of GCE and modified GCE electrodes of as a function of added SE with the cumulative concentrations of 4.7, 27.7, 74.7 nM, 0.31, 0.77, 3.07, 7.78, 30.82, and 77.92 μM, respectively. (B) Bar graphs of NCRs at three typical concentrations.

The *I*_ds_ represents the *I*_d_ after each addition, while *I*_ds,0_ shows the *I*_d_ before any addition. In Figure 8A, the NCRs of the bare GCE and different GCE modified gated sensors are shown. The bare GCE exhibited low sensitivity toward SE. However, after modification with MWCNTs, the sensitivity of the sensor increased significantly. This increase can be attributed to the larger surface area provided by MWCNTs, which offers more reaction sites for the oxidation of SE.^28^ This is evident in the CV of oxidation of SE using different electrodes, as shown in Figure 2A. The MWCNT-modified GCE electrode exhibits a significantly more intense oxidation peak for SE around 0.40 V compared to the bare GCE, which shows a negligible oxidation peak at the same bias. The GCE/MWCNT-gated OECT sensor also exhibited high transconductance (*g*_m_ ≈ 4 mS) at *V*_g_ = 0.5 V. The GCE/MWCNT/OMIP and GCE/MWCNT/DOMIP-gated sensors have slightly lower *g*_m_ than the GCE/MWCNT-gated device at *V*_g_ = 0.5 V, still enough to provide high sensitivity toward SE. The GCE/MWCNT/DOMIP electrode showed the highest sensitivity toward SE among all GCE and modified GCE electrodes, probably due to the electrostatic interaction between negatively charged PSS^–^ and positively charged SE in addition to the selective binding of SE into the imprinted cavities in the DOMIP film by shape complementarity and various noncovalent interactions, resulting in a lower limit of detection (LOD) of 0.31 μM. The response of OECT devices gated with GCE/MWCNT, GCE/MWCNT/OMIP, and GCE/MWCNT/DOMIP toward SE in high, medium, and low concentrations (0.77, 7.78, and 77.87 μM) are plotted in Figure 8B, showing that the GCE/MWCNT/DOMIP-gated sensor exhibits the highest sensitivity across all concentration ranges.

**Figure 9:**
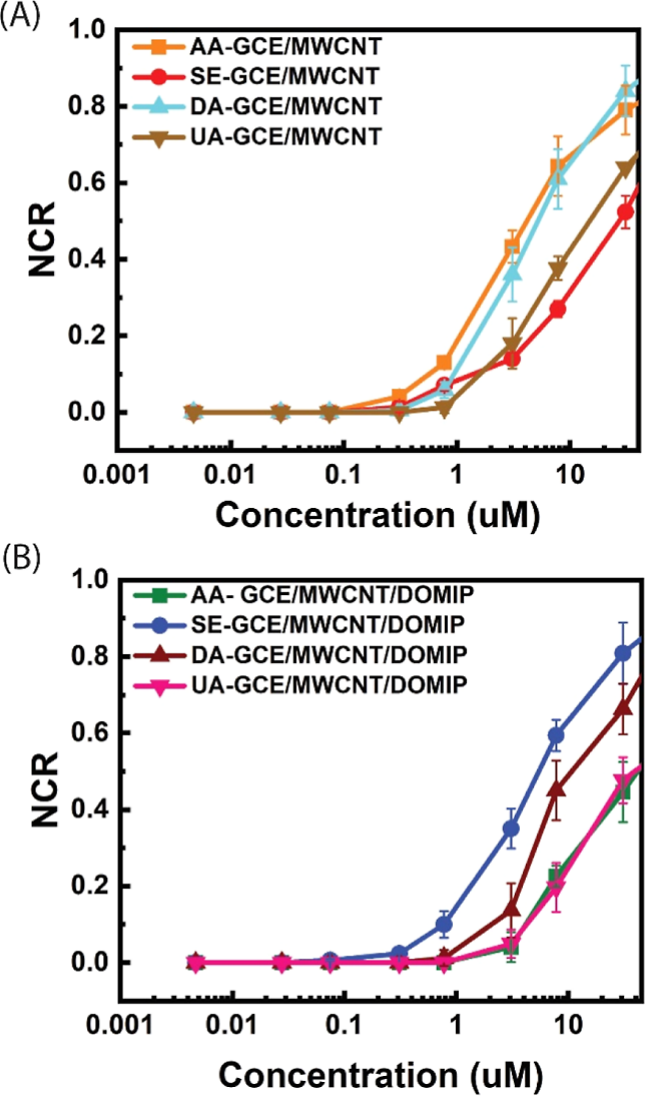
NCRs of SE and interferants including AA, DA and UA with the cumulative concentrations of 4.7, 27.7, 74.7 nM, 0.31, 0.77, 3.07, 7.78, 30.82, and 77.92 μM, using (A) GCE/MWCNT- and (B) GCE/MWCNT/DOMIP-gated OECTs.

Several biomolecules, including AA, UA, and DA, have chemical structures similar to that of SE.^16^ AA, UA, and DA exhibit lower oxidation potentials than SE, making them easier to oxidize.^31^ Particularly, DA’s chemical structure includes a catechol moiety, which is susceptible to oxidation, further facilitating its oxidation compared to SE.^32^ Therefore, when a sufficiently high *V*_g_ is applied, AA, UA, and DA can be oxidized on the gate electrode along with SE, thereby interfering with its detection.^31,32^ Therefore, real-time measurements were conducted using GCE/MWCNT- and GCE/MWCNT/DOMIP-gated OECT devices to compare the selectivity of OECT devices gated using the mentioned electrodes. Figure 9A shows the NCR of the GCE/MWCNT gated OECT sensor in the presence of SE, AA, DA, and UA. The highest sensitivity is observed toward AA and DA, and the lowest sensitivity is observed toward SE, which could be due to the higher oxidation potential of SE and its chemical structure, which is more resilient to oxidation than DA and AA.^31,32^

However, for the GCE/MWCNT/DOMIP gated OECT, the trend observed for GCE/MWCNT is reversed, and the doped MIP layer effectively blocks the negatively charged AA and UA due to its selective cavity and electrostatic repulsion from the negatively charged PSS^–^. In addition, the presence of PSS^–^, particularly its thiophene motifs, could also enhance the interaction between the SE and the DOMIP film. As shown in Figure 9B, the NCRs of the GCE/MWCNT/DOMIP-gated sensor in the low concentration range (0.31 and 0.78 μM) exhibit signals corresponding to SE additions, while displaying no detectable signals for AA and UA. The DOMIP layer also blocks most of DA; however, since DA is positively charged, it can be attracted by the negatively charged DOMIP film and enhances the response of the sensor toward DA, particularly at very high concentration where the diffusion of DA can be overwhelming. It should be noted that for DA, in the middle to high concentration range (7.8–7.78 μM), its NCR remains significantly lower than that of SE.

**Figure 10:**
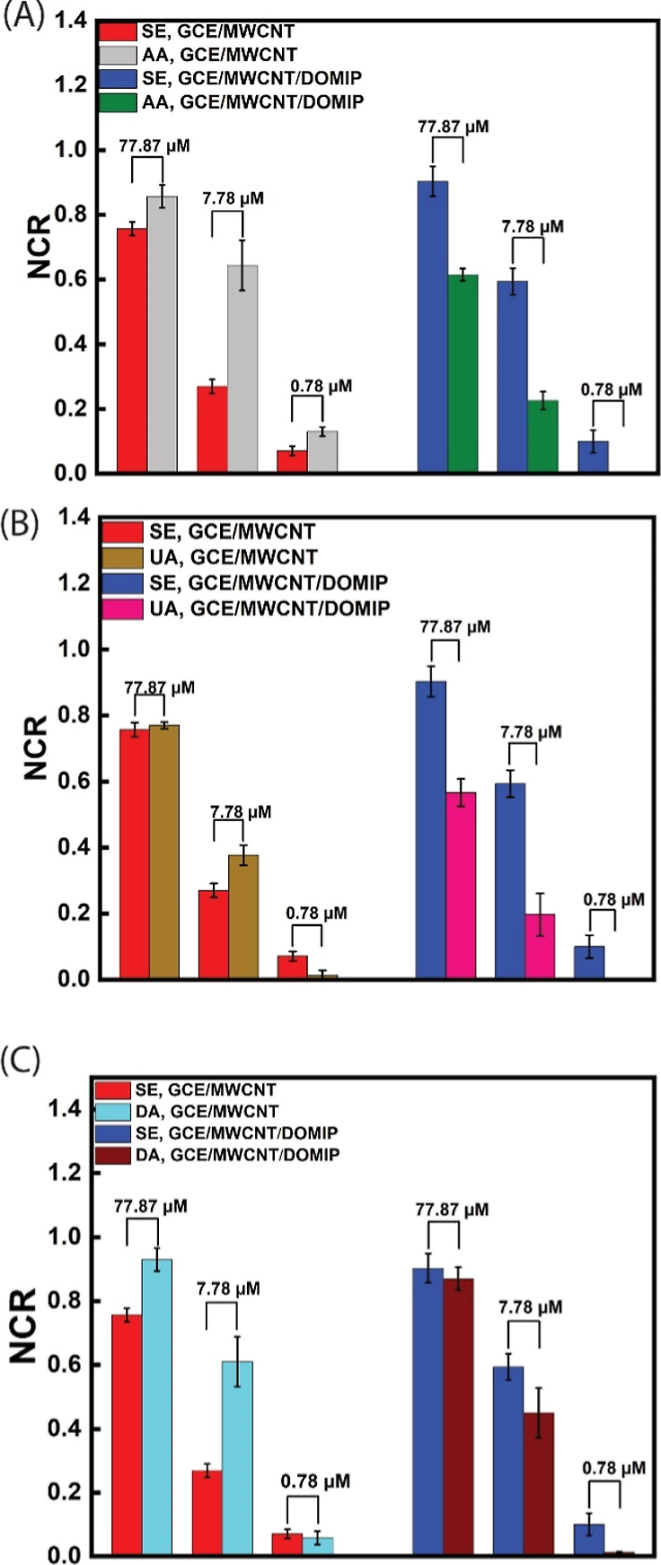
NCR of OECT sensors with GCE/MWCNT and GCE/MWCNT/DOMIP gate electrode for SE vs (A) AA, (B) UA, (C) DA in 0.78, 7.78, and 77.87 μM accumulative concentrations. Each error bar is derived from 3 devices.

Figure 10 shows bar graphs representing the NCR_SE_/NCR_interferants_ interferants ratios for both GCE/MWCNT and GCE/MWCNT/DOMIP-gated sensors at three distinct concentrations, chosen to represent the low (0.78 μM), middle (7.78 μM), and high concentration (77.87 μM) ranges. These bar graphs visualize the differences in selectivity between GCE/MWCNT-gated sensors and their GCE/MWCNT/DOMIP counterparts, highlighting the role of DOMIP in enhancing sensor selectivity. The GCE/MWCNT gated sensor exhibited lack of selectivity toward SE, as evidenced by the NCR_SE_/NCR_interferants_ ratios being consistently below one across all concentrations tested. However, the GCE/MWCNT/DOMIP-gated SE sensor in the lower concentration range (0.78 μM) exhibited a strong signal upon addition of SE while displaying no detectable signal for AA and UA. In the middle concentration range, the GCE/MWCNT/DOMIP gated sensor maintained very high selectivity toward SE, as evidenced by the NCR_SE_/NCR_Interferants_ ratios at 0.78 μM, that are 8.64, 7.10, and 2.56 for AA, UA, and DA, respectively. These results confirm the remarkable selectivity of the GCE/MWCNT/DOMIP gated sensor in the low and middle concentration ranges. However, as concentrations of analytes increase, the sensor’s selectivity reduces, as evidenced by a decline in NCR_SE_/NCR_Interferants_ ratios at 77.87 μM to 1.47, 1.59, and 1.03 for AA, UA, and DA, respectively. This could be due to saturation of selective cavities at higher concentrations.

These comparisons feature a noticeable increase in NCR for SE relative to the interferents when employing GCE/MWCNT/DOMIP-gated sensors, demonstrating the selectivity of the DOMIP layer across varying concentration regimes. The bar graphs clearly demonstrated a significantly higher selectivity for the GCE/MWCNT/DOMIP sensors compared with the GCE/MWCNT-gated sensors. Additionally, there was a trend of decreasing NCR_SE_/NCR_Interferents_ ratio and selectivity with increasing concentrations. These high selectivity results align with the normal concentration of SE in body fluids, which ranges from 0.1 to 1.1 μM in the plasma, and with elevated concentrations observed in carcinoid cancer situations, where the SE ranges from 4.5 to 9.5 μM.^33^

## Conclusions

In conclusion, this work developed a selective and sensitive sensor for the real-time detection of SE based on the use of OECTs. Serotonin-imprinted polymer films were synthesized electrochemically and coupled with multiwalled carbon nanotube (MWCNT) modifications to enhance both conductivity and selectivity. The redox reaction was established on the gate electrode to oxidize the SE, and the resulting faradaic current caused a shift in *V*_gm,eff_ and a step-like reduction in drain current (*I*_d_) after each addition of SE at different concentrations. The bare GCE exhibited low sensitivity toward SE. To address this, it was modified with MWCNTs to increase the surface area and conductivity of the electrode, leading to high sensitivity toward SE. However, the GCE/MWCNT-gated sensor showed low selectivity toward SE, displaying higher sensitivity toward interferants compared to SE. To improve selectivity, an MIP layer was doped with the negatively charged dopant PSS^–^ to enhance its selectivity through attractive electrostatic interactions with positively charged SE and repulsive forces against negatively charged UA and AA interferants. The DMIP layer was further oxidized in an alkaline solution to increase the surface area and reaction sites. The DOMIP layer demonstrated very high sensitivity toward SE and effectively blocked UA, AA, and DA interferences, reversing the trend observed with the GCE/MWCNT-gated sensors. The limit of detection (LOD) of the GCE/MWCNT/DOMIP-gated sensor is 0.31 μM, and it is capable of detecting SE concentrations within the biological range found in body fluids (0.1 to 1.1 μM in the plasma), as well as higher concentrations that serve as biomarkers for diseases such as carcinoid cancer. When compared with the existing literature, our findings align with the general trend of improving sensitivity and selectivity in OECT-based sensors while standing out due to the cost-effectiveness of the fabrication process. This work presents a simple alternative to antibodies and aptamers for electrode modification in electrochemical methods for the real-time measurement of small biomolecules. Additionally, this showcases how embedded dopants in MIP can significantly enhance sensitivity and selectivity. The simplicity of the fabrication process and its high sensitivity make it a promising candidate for practical applications in the monitoring of neurotransmitters in clinical diagnostics and other biomedical fields. Overall, this study paves the way for the development of more robust and accessible electrochemical sensors for the real-time detection of biomolecules.

## Supporting Information Available

Real-time drain current of OECT (PDF)

## References

[ref1] Friedlein J. T.; McLeod R. R.; Rivnay J. Device Physics of Organic Electrochemical Transistors. Org. Electron. 2018, 63, 398–414. 10.1016/j.orgel.2018.09.010.

[ref2] Kim S.-M.; Kim C.-H.; Kim Y.; Kim N.; Lee W.-J.; Lee E.-H.; Kim D.; Park S.; Lee K.; Rivnay J.; Yoon M.-H. Influence of PEDOT:PSS Crystallinity and Composition on Electrochemical Transistor Performance and Long-Term Stability. Nat. Commun. 2018, 9 (1), 3858 10.1038/s41467-018-06084-6. 30242224 PMC6155079

[ref3] Rivnay J.; Inal S.; Salleo A.; Owens R. M.; Berggren M.; Malliaras G. G. Organic Electrochemical Transistors. Nat. Rev. Mater. 2018, 3 (2), 17086 10.1038/natrevmats.2017.86.

[ref4] Tang K.; Turner C.; Case L.; Mehrehjedy A.; He X.; Miao W.; Guo S. Organic Electrochemical Transistor with Molecularly Imprinted Polymer-Modified Gate for the Real-Time Selective Detection of Dopamine. ACS Appl. Polym. Mater. 2022, 4 (4), 2337–2345. 10.1021/acsapm.1c01563.

[ref5] Gualandi I.; Tonelli D.; Mariani F.; Scavetta E.; Marzocchi M.; Fraboni B. Selective Detection of Dopamine with an All PEDOT:PSS Organic Electrochemical Transistor. Sci. Rep. 2016, 6 (1), 35419 10.1038/srep35419. 27739467 PMC5064404

[ref6] Fu Y.; Wang N.; Yang A.; Law H. K.; Li L.; Yan F. Highly Sensitive Detection of Protein Biomarkers with Organic Electrochemical Transistors. Adv. Mater. 2017, 29 (41), 1703787 10.1002/adma.201703787. 28922492

[ref7] Macchia E.; Romele P.; Manoli K.; Ghittorelli M.; Magliulo M.; Kovács-Vajna Z. M.; Torricelli F.; Torsi L. Ultra-Sensitive Protein Detection with Organic Electrochemical Transistors Printed on Plastic Substrates. Flexible Printed Electron. 2018, 3 (3), 034002 10.1088/2058-8585/aad0cb.

[ref8] Koklu A.; Wustoni S.; Musteata V.-E.; Ohayon D.; Moser M.; McCulloch I.; Nunes S. P.; Inal S. Microfluidic Integrated Organic Electrochemical Transistor with a Nanoporous Membrane for Amyloid-β Detection. ACS Nano 2021, 15 (5), 8130–8141. 10.1021/acsnano.0c09893. 33784064 PMC8158856

[ref9] Tang H.; Yan F.; Lin P.; Xu J.; Chan H. L. W. Highly Sensitive Glucose Biosensors Based on Organic Electrochemical Transistors Using Platinum Gate Electrodes Modified with Enzyme and Nanomaterials. Adv. Funct. Mater. 2011, 21 (12), 2264–2272. 10.1002/adfm.201002117.

[ref10] Liao C.; Mak C.; Zhang M.; Chan H. L. W.; Yan F. Flexible Organic Electrochemical Transistors for Highly Selective Enzyme Biosensors and Used for Saliva Testing. Adv. Mater. 2015, 27 (4), 676–681. 10.1002/adma.201404378. 25469658

[ref11] Kergoat L.; Piro B.; Simon D. T.; Pham M.; Noël V.; Berggren M. Detection of Glutamate and Acetylcholine with Organic Electrochemical Transistors Based on Conducting Polymer/Platinum Nanoparticle Composites. Adv. Mater. 2014, 26 (32), 5658–5664. 10.1002/adma.201401608. 24924118

[ref12] Nielsen C. B.; Giovannitti A.; Sbircea D.-T.; Bandiello E.; Niazi M. R.; Hanifi D. A.; Sessolo M.; Amassian A.; Malliaras G. G.; Rivnay J.; McCulloch I. Molecular Design of Semiconducting Polymers for High-Performance Organic Electrochemical Transistors. J. Am. Chem. Soc. 2016, 138 (32), 10252–10259. 10.1021/jacs.6b05280. 27444189 PMC4991841

[ref13] Nichkova M. I.; Huisman H.; Wynveen P. M.; Marc D. T.; Olson K. L.; Kellermann G. H. Evaluation of a Novel ELISA for Serotonin: Urinary Serotonin as a Potential Biomarker for Depression. Anal. Bioanal. Chem. 2012, 402 (4), 1593–1600. 10.1007/s00216-011-5583-1. 22160204

[ref14] Kema I. P.; De Vries E. G. E.; Muskiet F. A. J. Clinical Chemistry of Serotonin and Metabolites. J. Chromatogr. B 2000, 747 (1–2), 33–48. 10.1016/S0378-4347(00)00341-8. 11103898

[ref15] Feldman J. M. Urinary Serotonin in the Diagnosis of Carcinoid Tumors. Clin. Chem. 1986, 32 (5), 840–844. 10.1093/clinchem/32.5.840. 2421946

[ref16] Sharma S.; Singh N.; Tomar V.; Chandra R. A Review on Electrochemical Detection of Serotonin Based on Surface Modified Electrodes. Biosens. Bioelectron. 2018, 107, 76–93. 10.1016/j.bios.2018.02.013. 29448224

[ref17] Zhang S.; Ling P.; Chen Y.; Liu J.; Yang C. 2D/2D Porous Co3O4/rGO Nanosheets Act as an Electrochemical Sensor for Voltammetric Tryptophan Detection. Diamond Relat. Mater. 2023, 135, 109811 10.1016/j.diamond.2023.109811.

[ref18] Wu B.; Xiao L.; Zhang M.; Yang C.; Li Q.; Li G.; He Q.; Liu J. Facile Synthesis of Dendritic-like CeO2/rGO Composite and Application for Detection of Uric Acid and Tryptophan Simultaneously. J. Solid State Chem. 2021, 296, 122023 10.1016/j.jssc.2021.122023.

[ref19] Dankoski E. C.; Wightman R. M. Monitoring Serotonin Signaling on a Subsecond Time Scale. Front. Integr. Neurosci. 2013, 7. 10.3389/fnint.2013.00044. PMC3672682 23760548

[ref20] Dunham K. E.; Venton B. J. Improving Serotonin Fast-Scan Cyclic Voltammetry Detection: New Waveforms to Reduce Electrode Fouling. Analyst 2020, 145 (22), 7437–7446. 10.1039/D0AN01406K. 32955048 PMC7655692

[ref21] Lim S. G.; Seo S. E.; Park S. J.; Kim J.; Kim Y.; Kim K. H.; An J. E.; Kwon O. S. Real-Time Monitoring of Serotonin with Highly Selective Aptamer-Functionalized Conducting Polymer Nanohybrids. Nano Convergence 2022, 9 (1), 31 10.1186/s40580-022-00325-7. 35829851 PMC9279540

[ref22] Ahmad H. M. N.; Andrade A.; Song E. Continuous Real-Time Detection of Serotonin Using an Aptamer-Based Electrochemical Biosensor. Biosensors 2023, 13 (11), 983 10.3390/bios13110983. 37998158 PMC10669129

[ref23] Cuniberto E.; Huang Z.; Ward M. D.; Shahrjerdi D. Unraveling the Complex Electrochemistry of Serotonin Using Engineered Graphitic Sensors. Analyst 2022, 148 (1), 105–113. 10.1039/D2AN01451C. 36412489

[ref24] Chavan S. G.; Rathod P. R.; Koyappayil A.; Hwang S.; Lee M.-H. Recent Advances of Electrochemical and Optical Point-of-Care Biosensors for Detecting Neurotransmitter Serotonin Biomarkers. Biosens. Bioelectron. 2025, 267, 116743 10.1016/j.bios.2024.116743. 39270361

[ref25] Bai L.; Elósegui C. G.; Li W.; Yu P.; Fei J.; Mao L. Biological Applications of Organic Electrochemical Transistors: Electrochemical Biosensors and Electrophysiology Recording. Front. Chem. 2019, 7, 313 10.3389/fchem.2019.00313. 31134185 PMC6514146

[ref26] Sasso L.; Heiskanen A.; Diazzi F.; Dimaki M.; Castillo-León J.; Vergani M.; Landini E.; Raiteri R.; Ferrari G.; Carminati M.; Sampietro M.; Svendsen W. E.; Emnéus J. Doped Overoxidized Polypyrrole Microelectrodes as Sensors for the Detection of Dopamine Released from Cell Populations. Analyst 2013, 138 (13), 3651 10.1039/c3an00085k. 23628978

[ref27] Davis S. E.; Korich A. L.; Ramsson E. S. Enhancement of Fast Scan Cyclic Voltammetry Detection of Dopamine with Tryptophan-Modified Electrodes. PLoS One 2020, 15 (7), e0235407 10.1371/journal.pone.0235407. 32649670 PMC7351191

[ref28] Zheng D.; Vashist S.; Dykas M.; Saha S.; Al-Rubeaan K.; Lam E.; Luong J.; Sheu F.-S. Graphene versus Multi-Walled Carbon Nanotubes for Electrochemical Glucose Biosensing. Materials 2013, 6 (3), 1011–1027. 10.3390/ma6031011. 28809354 PMC5512961

[ref29] Koluaçık E.; Karabiberoğlu S. ¸. U.; Dursun Z. Electrochemical Determination of Serotonin Using Pre-treated Multi-walled Carbon Nanotube-polyaniline Composite Electrode. Electroanalysis 2018, 30 (12), 2977–2987. 10.1002/elan.201800588.

[ref30] Husain A.; Mahajan D. K. Effect of Multi-Walled Carbon Nanotubes on DC Electrical Conductivity and Acetone Vapour Sensing Properties of Polypyrrole. Carbon Trends 2022, 9, 100193 10.1016/j.cartre.2022.100193.

[ref31] Kim S. K.; Kim D.; Jeon S. Electrochemical Determination of Serotonin on Glassy Carbon Electrode Modified with Various Graphene Nanomaterials. Sens. Actuators, B 2012, 174, 285–291. 10.1016/j.snb.2012.08.034.

[ref32] Sulzer D.; Zecca L. Intraneuronal Dopamine-Quinone Synthesis: A Review. Neurotoxic. Res. 1999, 1 (3), 181–195. 10.1007/BF03033289. 12835101

[ref33] Gut P.; Czarnywojtek A.; Sawicka-Gutaj N.; Ruchała M. Assessment of Serotonin Concentration in Patients with Small-Intestine Neuroendocrine Neoplasm and Carcinoid Syndrome Treated with Somatostatin Analogues. Polym. Arch. Int. Med. 2020, 130, 903–905. 10.20452/pamw.15504. 32643913

