# Supplementary material for: Selective and Sensitive OECT Sensors with Doped MIP-Modified GCE/MWCNT Gate Electrodes for Real-Time Detection of Serotonin
Source: ACS Omega. 2025 Jan 24;10(4):4154–62. doi: 10.1021/acsomega.4c10918 (PMC11800035; doi:10.1021/acsomega.4c10918)
Supplement: Supplementary file 1 — ao4c10918_si_001.pdf [file ao4c10918_si_001.pdf]

Supporting Information

Selective and sensitive OECT sensor with doped MIP-modified GCE/MWCNT gate electrode for real-time detection of serotonin

Amin Mehrehjedy,[a] *Jack Eaton,*[a,b] *Kan Tang,*[a] *Saroj Upreti,*[c] *Aries Sanders,*[d] *Vincent LaRoux,*[a] *Xiaodan Gu,*[c] *Xuyang He,*[b] *Song Guo**[,a]

a. Department of Chemistry and Biochemistry, School of Mathematics and Natural Sciences, The University of Southern Mississippi, Hattiesburg, MS, 39406, United States

b. School of Criminal Justice, Forensic Science, and Security, The University of Southern Mississippi, Hattiesburg, Mississippi 39406, United States

c. School of Polymer Science and Engineering, The University of Southern Mississippi, Hattiesburg, MS 39406, United States

d. Department of Chemistry, The University of Arkansas – Fort Smith, Fort Smith, Arkansas 72913, United States

Email: song.guo@usm.edu

(A)

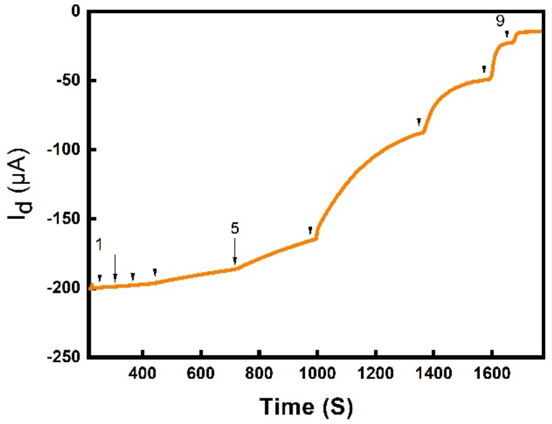

0
-50
-100
-150
-200
-250
400
600
800
1000
1200
1400
1600
Time (S)
I_d (µA)
1
5
9

(B)

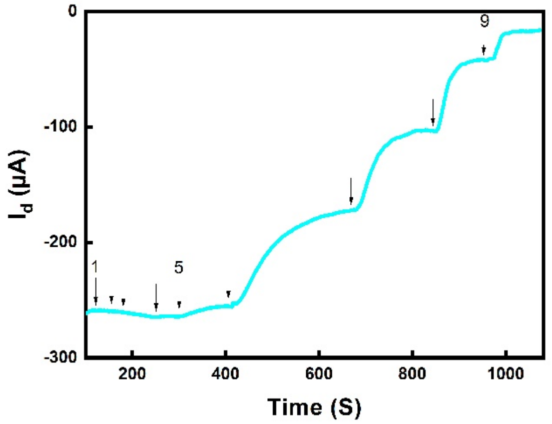

0
-100
-200
-300
200
400
600
800
1000
Time (S)
I_d (µA)
1
5
9

(C)

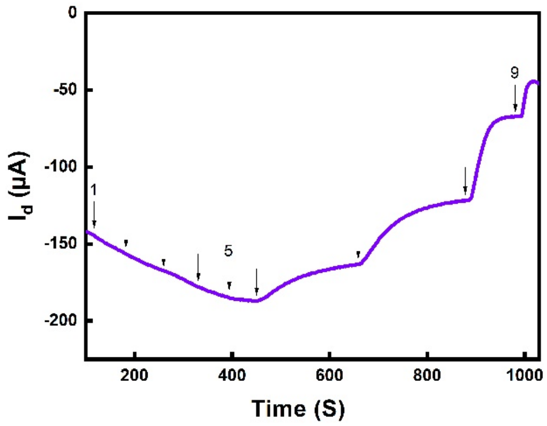

0
-50
-100
-150
-200
200
400
600
800
1000
Time (S)
1
5
9

(D)

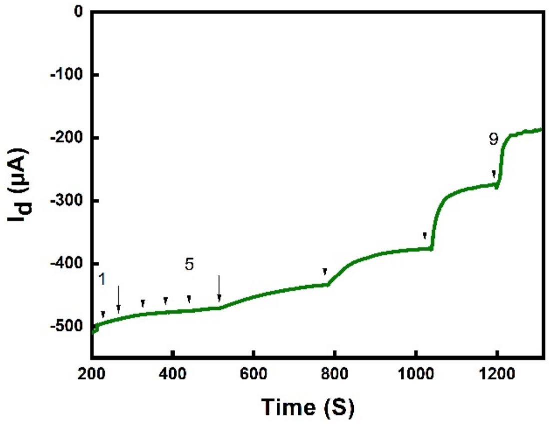

0
-100
-200
-300
-400
-500
200
400
600
800
1000
1200
Time (S)
1
5
9

(E)

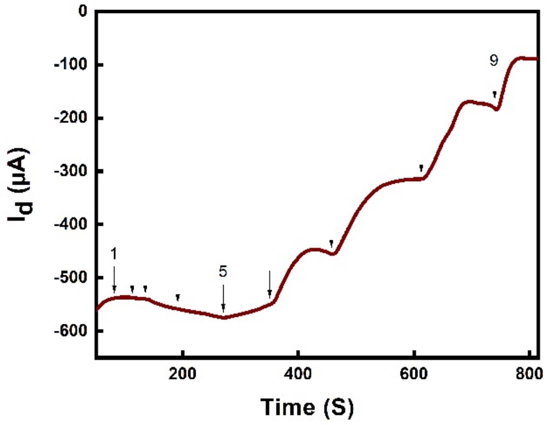

0
-100
-200
-300
-400
-500
-600
200
400
600
800
Time (S)
1
5
9

(F)

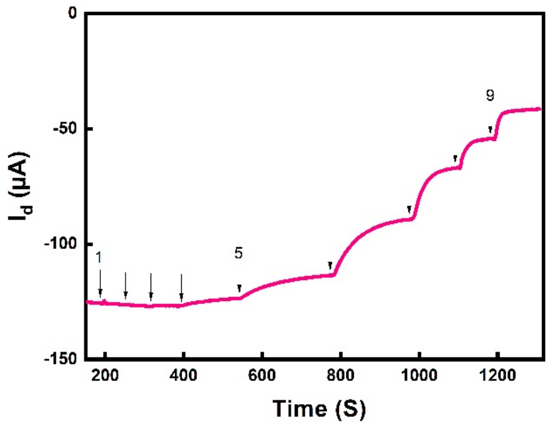

Figure S1. Real-time trace of I_{ds} of OECT sensors sequential additions of interferants in PBS buffer. OECT sensor gated with GCE/MWCNT (A) AA, (B) DA, (C) UA and gated with GCE/MWCNT/DOMIP (D) AA, (E) DA, (F) UA.
